# Supplementary figures and images for: Perceptions and predictors of COVID-19 vaccine hesitancy among healthcare providers across five countries in sub-Saharan Africa
Source: PLOS Glob Public Health. 2025 Feb 21;5(2):e0003956. doi: 10.1371/journal.pgph.0003956 (PMC11844854; doi:10.1371/journal.pgph.0003956)

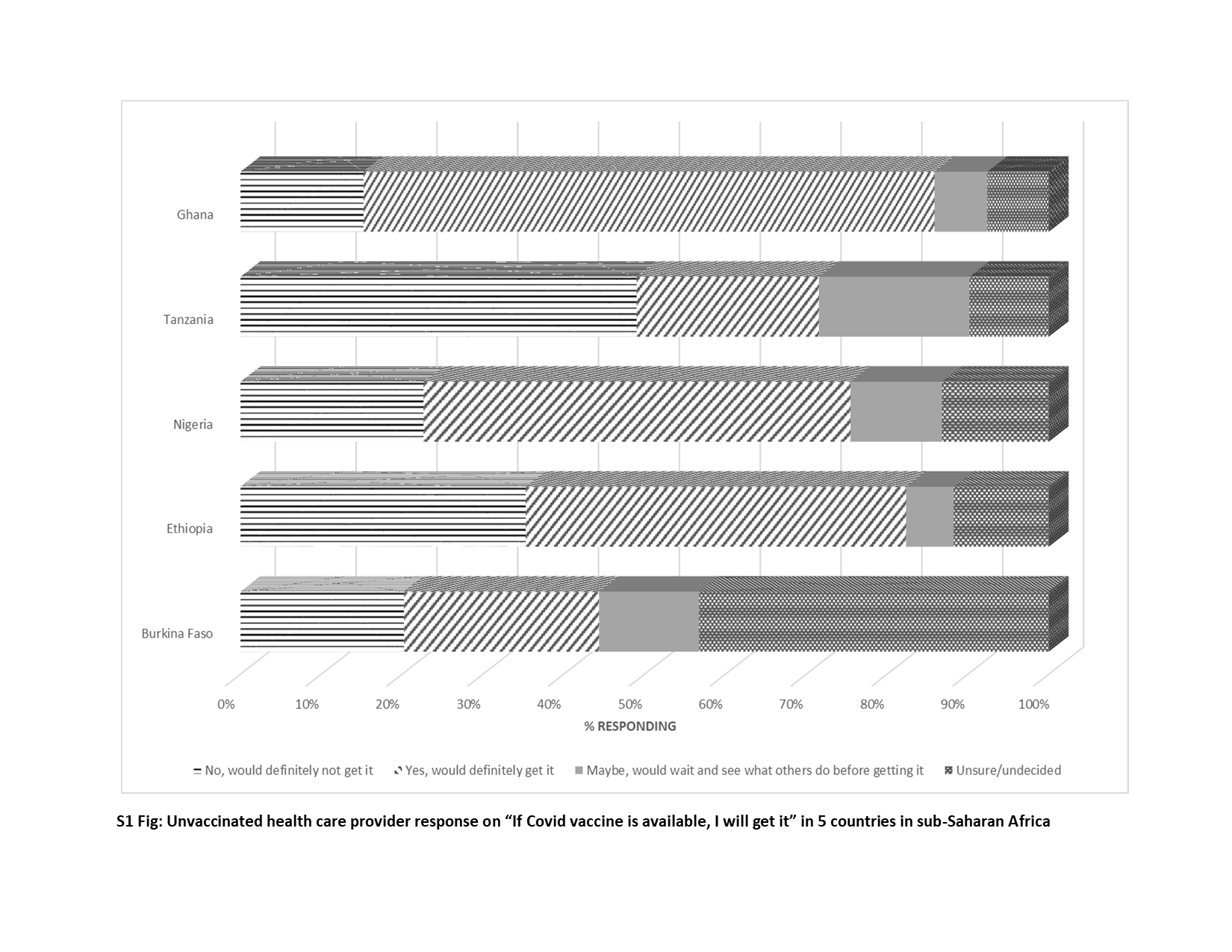

Supplement: S1 Fig — (TIF) [file pgph.0003956.s001.tif]

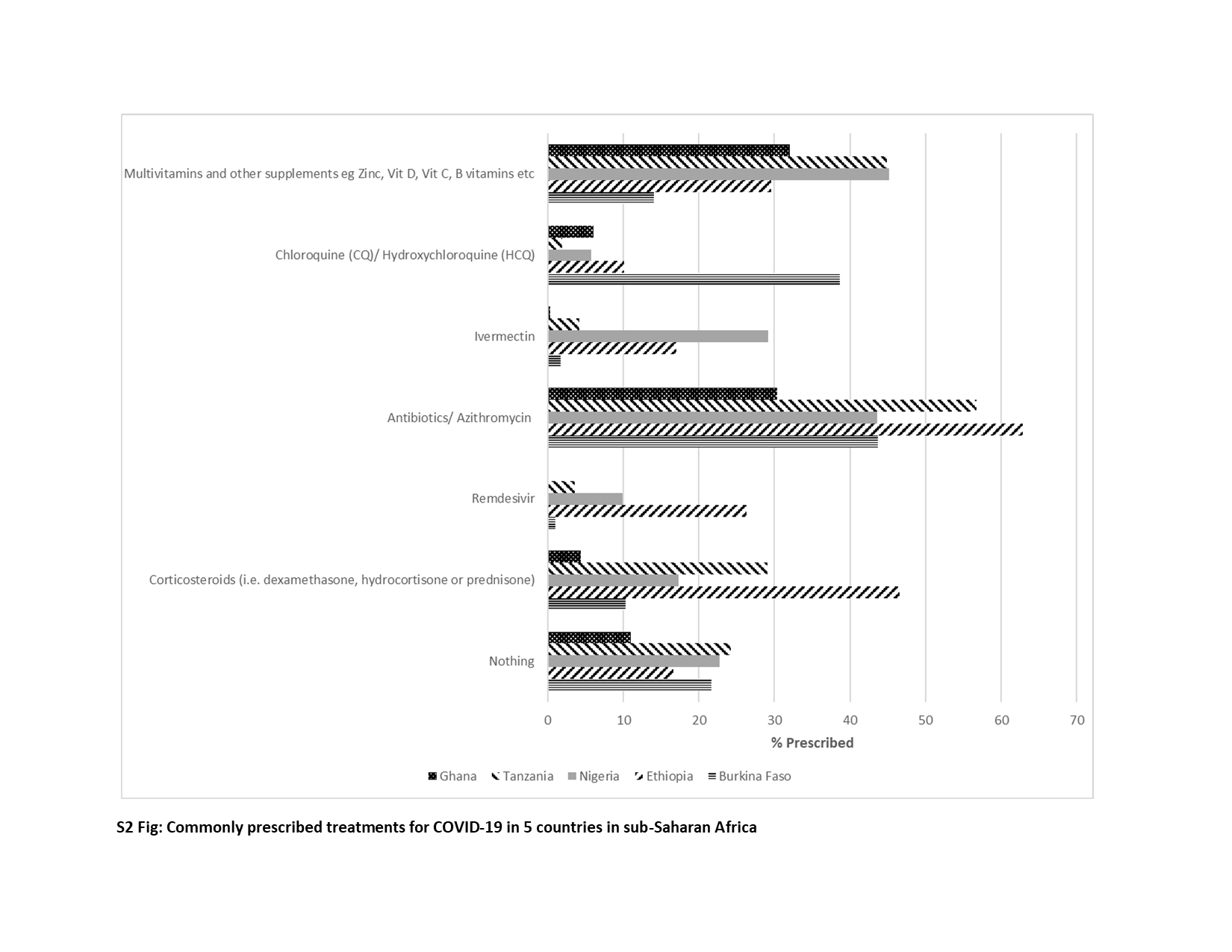

Supplement: S2 Fig — (TIF) [file pgph.0003956.s002.tif]
