# Supplementary material for: Perceptions and predictors of COVID-19 vaccine hesitancy among healthcare providers across five countries in sub-Saharan Africa
Source: PLOS Glob Public Health. 2025 Feb 21;5(2):e0003956. doi: 10.1371/journal.pgph.0003956 (PMC11844854; doi:10.1371/journal.pgph.0003956)
Supplement: S1 Table — (DOCX) [file pgph.0003956.s003.docx]

**S Table 1: Proportion of participants participating in survey rounds**

| **Proportion of participants participating in survey rounds** |  | **Country/Region** | | | | |  |
| --- | --- | --- | --- | --- | --- | --- | --- |
|  |  | **Burkina Faso** | **Ethiopia** | **Nigeria** | **Tanzania** | **Ghana** | **Total** |
| Round 1 | n | 222 | 208 | \| 147 \| \| --- \| | 0 | 0 | 577 |
|  | % | 74.00 | 75.09 | 47.12 | 0 | 0 | 38.49 |
| Round 2 | n | 78 | 69 | 165 | 310 | 300 | 922 |
|  | % | 26.00 | 24.91 | 52.88 | 100 | 100 | 61.51 |
| **Total** |  | **300** | **277** | **312** | **310** | **300** | **1499** |
