# Supplementary material for: Perceptions and predictors of COVID-19 vaccine hesitancy among healthcare providers across five countries in sub-Saharan Africa
Source: PLOS Glob Public Health. 2025 Feb 21;5(2):e0003956. doi: 10.1371/journal.pgph.0003956 (PMC11844854; doi:10.1371/journal.pgph.0003956)
Supplement: S1 Text — (PDF) [file pgph.0003956.s004.pdf]

ARISE Network COVID-19 Round 2 Healthcare Provider Survey

To be administered over the phone to healthcare providers.

INTERVIEWER QUESTIONS

Interview date and time to be automatically coded by ODK.

Question	Response
Country name	1 = Burkina Faso<br>2 = Ethiopia<br>3 = Nigeria<br>4 = Tanzania DSM<br>5 = Ghana
Data collector's code	
Participant initials (first initial of first, middle and last name)	
Was this participant included in the round 1 survey?<br>*(omit this question for Tanzania and Ghana)*<br><br>*If Yes, please input the ID they were assigned in the last survey in the next field. If they are a new participant, assign them a new ID.*	0=No<br>1=Yes
Participant identification number	
Phone number called	
Was the phone call answered?	0=No<br>1=Yes
If No, how many times have you tried to reach this phone number? *Note to interviewer: each phone number should be tried three times on separate days.*	0=This was the first attempt<br>1=This was the second attempt<br>2=This was the third (final) attempt<br>99= Don't know
If Yes, what was the outcome of the call?	0=Participant unavailable now but requested a call-back<br>1=Participant unavailable now but did not want to be called back<br>2=Participant refused to continue the phone conversation or hung up<br>3=Participant agreed to proceed with the screening questions<br>4= Other (specify)
Specify other	

PARTICIPANT QUESTIONS

<u>Screening Questions</u>

Question	Responses
Do you currently work in a health care setting?<br><br>*If No, thank the participant and end the interview.*	0=No<br>1=Yes
What is your occupation?	1 = Doctor

If response is 1-5 proceed with the survey<br>*If response is other, end survey*	2 = Nurse<br>3 = Clinical officer<br>4 = Community health worker<br>5= Medical Assistant<br>96= Other (specify)

Consent

Does the participant consent to participate in the survey?	0 = No<br>1 = Yes<br><br>*If the response is No, terminate the interview*

1. Descriptive Characteristics

**Question**	**Response**
1.1.1 What state/region/province do you currently live in?	*Insert dropdown list for each country.*
1.1.2 What is your age in years?<br>*Refer to completed years*	__ __ years
1.1.3 What is your gender?	0 = Female<br>1 = Male
1.1.4 What type of healthcare facility do you work in?	1 = Government hospital/clinic<br>2 = Private hospital/clinic<br>3 = Health outpost/CHPS compounds<br>96 = Other (specify)<br>97 = Don't know<br>99 = Refuse to answer
1.1.5 What is your religion?	0 = None<br>1 = Catholic<br>2 = Muslim<br>3 = Orthodox Christian<br>4 = Protestant/Other Christian<br>96 = Other (specify)<br>99 = Refuse to answer

2. Perceptions of COVID-19

**Question**	**Responses**
2.1.1 Are you concerned about the spread of COVID-19?	0 = No<br>1 = Yes<br>97 = Don't know<br>99 = Refuse to answer
2.1.2 In your opinion, what do you think is your level of risk of exposure to COVID-19?	0 = No risk<br>1 = Low risk

	2 = High risk<br>3 = Very high risk<br>97 = Don't know<br>99 = Refuse to answer

3. Mental Health and COVID-19

**Question**	**Responses**
3.1.1. Over the last two weeks, how often have you been bothered by the following problems?	
a. Feeling nervous, anxious or on edge	0 = Not at all<br>1 = Several days<br>2 = More than half the days<br>3 = Nearly everyday<br>97 = Don't know<br>99 = Refuse to answer
b. Not being able to stop or control worrying	0 = Not at all<br>1 = Several days<br>2 = More than half the days<br>3 = Nearly everyday<br>97 = Don't know<br>99 = Refuse to answer
c. Feeling down, depressed or hopeless	0 = Not at all<br>1 = Several days<br>2 = More than half the days<br>3 = Nearly everyday<br>97 = Don't know<br>99 = Refuse to answer
d. Little interest or pleasure in doing things	0 = Not at all<br>1 = Several days<br>2 = More than half the days<br>3 = Nearly everyday<br>97 = Don't know<br>99 = Refuse to answer

4. COVID-19 in the Workplace

**Question**	**Responses**
4.1.1. Which of the following measures are currently being implemented in your workplace to prevent the spread of COVID-19?	
a) Wearing masks	0 = No<br>1 = Yes<br>97 = Don't know<br>99 = Refuse to answer
b) Using personal protective equipment (PPE) such as gowns, goggles, shields, etc.	0 = No<br>1 = Yes<br>97 = Don't know<br>99 = Refuse to answer

c) Hand washing with water and soap	0 = No<br>1 = Yes<br>97 = Don’t know<br>99 = Refuse to answer
d) Keeping sufficient distance between patients in the waiting area	0 = No<br>1 = Yes<br>97 = Don’t know<br>99 = Refuse to answer
e) Presence of sanitizers or hand washing station in all service delivery points	0 = No<br>1 = Yes<br>97 = Don’t know<br>99 = Refuse to answer
f) Regular cleaning and decontamination/disinfection of public areas and offices	0 = No<br>1 = Yes<br>97 = Don’t know<br>99 = Refuse to answer
g) Temperature measurement/check high temperatures (37 degrees Celsius)	0 = No<br>1 = Yes<br>97 = Don’t know<br>99 = Refuse to answer
h) Other (specify)	0 = No<br>1 = Yes<br>97 = Don’t know<br>99 = Refuse to answer
4.1.2. Is COVID-19 testing available at your workplace for the public or patients?<br><br>*If no, skip to 4.1.4.*	0 = No<br>1 = Yes<br>97 = Don’t know<br>99 = Refuse to answer
4.1.2b Is the available COVID-19 testing free or paid?	0= Yes, free testing<br>1= Yes, paid testing<br>97= Don't know<br>99= Refuse to answer
4.1.3. What type of COVID-19 tests are available at your health centre?<br><br>*Select all that apply.*	1 = PCR test (nasal swab or saliva)<br>2 = Antigen test (rapid nasal swab test)<br>96 = Other (specify)<br>97 = Don’t know
4.1.4. Have you ever been tested for COVID-19?<br><br>*If no, skip to 4.1.6.*	0 = No<br>1 = Yes<br>97 = Don’t know<br>99 = Refuse to answer
4.1.5. Have you ever tested positive for COVID-19?	0 = No<br>1 = Yes<br>97 = Don’t know<br>99 = Refuse to answer
4.1.6. Have you cared for any patients with COVID-19 in your workplace or in the community?	0 = Never<br>1 = Yes, in the past 1 month<br>2 = Yes, over one month ago<br>97 = Don’t know<br>99 = Refuse to answer
4.1.7. Does your workplace have formulated policies or guidance related to COVID-19?	0 = No<br>1 = Yes

If no, skip to question 4.1.9.	97 = Don't know<br>99 = Refuse to answer
4.1.9. Have you received any formal training or orientation from your office or employer on the following topics in the past 6 months?	
a. Natural course of COVID-19 disease, including symptoms, signs and clinical markers	0 = No<br>1 = Yes<br>97 = Don't know<br>99 = Refuse to answer
b. Management and treatment of COVID-19	0 = No<br>1 = Yes<br>97 = Don't know<br>99 = Refuse to answer
c. How the COVID-19 vaccines work, risk of potential side effects, and importance of vaccination	0 = No<br>1 = Yes<br>97 = Don't know<br>99 = Refuse to answer
d. Managing COVID-19 vaccination programs, including registration, administering vaccines in the facility or in communities, and collecting data on vaccination	0 = No<br>1 = Yes<br>97 = Don't know<br>99 = Refuse to answer
4.1.10. What kind of treatment is your facility currently recommending for treating COVID-19 cases?<br><br>*Select all that apply.*	0 = Nothing<br>1 = Corticosteroids (i.e. dexamethasone, hydrocortisone or prednisone)<br>2 = Remdesivir<br>3 = Antibiotics/ Azithromycin<br>4 = Ivermectin<br>5 =Chloroquine (CQ)/ Hydroxychloroquine (HCQ)<br>6= Multivitamins and other supplements eg Zinc, Vit D, Vit C, B vitamins etc<br>96 = Other (specify)<br>97 = Don't know
4.1.11. How is COVID-19 currently impacting the following healthcare services?	
a. Childhood immunization	0 = No impact<br>1 = Services are paused<br>2 = Services are operating at minimum level/only provided for emergencies<br>97 = Don't know/Don't work in the unit<br>98= Not applicable/Service not available at facility<br>99 = Refuse to answer
b. Vitamin A supplementation for children	0 = No impact<br>1 = Services are paused<br>2 = Services are operating at minimum level/only provided for emergencies<br>97 = Don't know/Don't work in the unit<br>98= Not applicable/Service not available at facility

	99 = Refuse to answer
d. Management of child malnutrition (e.g. severe acute malnutrition)	0 = No impact<br>1 = Services are paused<br>2 = Services are operating at minimum level/only provided for emergencies<br>97 = Don't know/Don't work in the unit<br>98= Not applicable/Service not available at facility<br>99 = Refuse to answer
e. Antenatal care for pregnant women	0 = No impact<br>1 = Services are paused<br>2 = Services are operating at minimum level/only provided for emergencies<br>97 = Don't know/Don't work in the unit<br>98= Not applicable/Service not available at facility<br>99 = Refuse to answer
f. Iron and folic acid for pregnant women	0 = No impact<br>1 = Services are paused<br>2 = Services are operating at minimum level/only provided for emergencies<br>97 = Don't know/Don't work in the unit<br>98= Not applicable/Service not available at facility<br>99 = Refuse to answer
g. HIV treatment services	0 = No impact<br>1 = Services are paused<br>2 = Services are operating at minimum level/only provided for emergencies<br>97 = Don't know/Don't work in the unit<br>98= Not applicable/Service not available at facility<br>99 = Refuse to answer
h. TB treatment services	0 = No impact<br>1 = Services are paused<br>2 = Services are operating at minimum level/only provided for emergencies<br>97 = Don't know/Don't work in the unit<br>98= Not applicable/Service not available at facility<br>99 = Refuse to answer
i. Sexual and reproductive health including family planning	0 = No impact<br>1 = Services are paused<br>2 = Services are operating at minimum level/only provided for emergencies<br>97 = Don't know/Don't work in the unit<br>98= Not applicable/Service not available at facility<br>99 = Refuse to answer

j. Surgeries	0 = No impact<br>1 = Services are paused<br>2 = Services are operating at minimum level/only provided for emergencies<br>97 = Don't know/Don't work in the unit<br>98= Not applicable/Service not available at facility<br>99 = Refuse to answer
**4.2 Questions Assessing Stigma**	
4.2.1. In the last 6 months, have you experienced any of the following due to your profession?	
a. Social avoidance or rejection	0 = No<br>1 = Yes<br>97 = Don't know<br>99 = Refuse to answer
b. Denial of healthcare, education, housing or employment	0 = No<br>1 = Yes<br>97 = Don't know<br>99 = Refuse to answer
c. Physical violence	0 = No<br>1 = Yes<br>97 = Don't know<br>99 = Refuse to answer
d. Congratulations or acknowledgment	0 = No<br>1 = Yes<br>97 = Don't know<br>99 = Refuse to answer

5. Vaccines

**Question**	**Responses**
**5.1. Vaccine knowledge, attitudes, practices**	
5.1.1. In general, do you believe that vaccines are safe?	0=No<br>1=Yes<br>97 = Don't know<br>99=Refuse to answer
5.1.2. Has your religious leader ever said anything about vaccination in general?	0=No<br>1=Yes, something positive<br>2=Yes, something negative<br>3=I don't have a religious leader<br>97 = Don't know<br>99 = Refuse to answer
5.1.3. Is a COVID-19 vaccine available in [COUNTRY]?	0=No<br>1=Yes<br>97 = Don't know<br>99 = Refuse to answer
5.1.4. Has the COVID-19 vaccine been made available to you as a healthcare provider?	0=No<br>1=Yes

	97 = Don't know<br>99 = Refuse to answer
5.1.5. Have you or any of your colleagues received the COVID-19 vaccination?	0=No<br>1=Yes, I have been vaccinated<br>2=I know someone who has been vaccinated<br>97 = Don't know<br>99 = Refuse to answer
5.2. **COVID vaccine awareness and perception**	
5.2.1. In general, how safe do you think COVID-19 vaccines are?	1=Very safe<br>2=Somewhat safe<br>3=Not very safe<br>4=Not at all safe<br>97 = Don't know<br>99 = Refuse to answer
5.2.2. In general, how effective do you think the COVID-19 vaccines are in preventing COVID infection?	1=Very effective<br>2=Somewhat effective<br>3=Not very effective<br>4=Not effective at all<br>97 = Don't know<br>99 = Refuse to answer
5.2.3. Does the COVID-19 vaccine have any side effects that you are aware of?<br>*(Do not read answer choices aloud; code responses and/or enter "other")*<br>5.2.4. *(Select all that apply)*	0=No side effects<br>1=Fever<br>2=Body ache, including arm pain<br>3=Nausea<br>4=Tiredness/exhaustion<br>96= Other (specify)<br>97 = Don't know<br>99 = Refuse to answer
5.2.5. How much do you agree with this statement?<br><br>"If a vaccine for COVID-19 were available now, I would definitely get it."<br><br>*Skip if 5.1.5 = 1*	0=No, would definitely not get it<br>1=Yes, would definitely get it<br>2=Maybe, would wait and see what others do before getting it<br>3=Unsure/undecided<br>99 = Refuse to answer
5.2.6. Which of the following are reasons that you *would/did* get the COVID-19 vaccine?<br>*If 5.2.5 = Yes, Maybe, or Unsure/undecided, or if 5.1.5 = 1*	
a) To keep myself or my family safe	0=Disagree / No<br>1=Agree / Yes<br>97 = Don't know<br>99 = Refuse to answer
b) To keep my patients and colleagues safe	0=Disagree / No<br>1=Agree / Yes<br>97 = Don't know<br>99 = Refuse to answer
c) Because my friend/family suggested I get it	0=Disagree / No<br>1=Agree / Yes<br>97 = Don't know<br>99 = Refuse to answer
d) Because my superiors/managers instruct me to get it	0=Disagree / No<br>1=Agree / Yes<br>97 = Don't know<br>99 = Refuse to answer
e) There are additional reasons I would/did get the vaccine	0=Disagree / No<br>1=Agree / Yes *(Specify)*_______

5.2.7. What are the reasons why you would *not* get the COVID-19 vaccine?<br><br>*If 5.2.5 = No or Unsure/undecided*<br><br>*(Do not read answer choices aloud; code responses and/or enter "other")*<br>*(Select all that apply)*	1=Do not think it is needed<br>2=Do not think I am at risk of getting COVID<br>3=Do not think the vaccine is effective against COVID-19<br>4= Heard/read negative media reports<br>5=Do not think the vaccine is safe/ It was developed too fast<br>6= Concerned about side effects<br>7= Fear of getting an unlicensed / experimental vaccine, fear that people here will get worse quality vaccines<br>8=Fear getting COVID-19 disease from the vaccine<br>9=Fear getting other illnesses / autism from the vaccine<br>10= Fear the vaccine will cause infertility / sterilization / population control<br>11=Religious reasons/church or religion advises against<br>12=Fear of microchipping<br>13=Fear of New World Order<br>14= Had a bad experience or reaction with previous vaccinations<br>15=Concerned because I have a chronic condition e.g. diabetes, hypertension and not sure it is safe for people with my condition<br>16=Personal liberty / do not want bodily intrusion<br>96= Other (specify)<br>97 = Don't know<br>99 = Refuse to answer
**5.3. Willingness to get the COVID vaccine**	
5.3.1. I am going to list several statements about the COVID-19 vaccine. Some are true and some are false. Do you believe the following statements about the COVID-19 vaccine are true or false?	
a) Vaccine trial participants have died after taking the vaccine	1=True / Yes<br>0=False / No<br>97 = Don't know<br>99 = Refuse to answer
b) Side effects from the COVID-19 vaccine are usually mild and temporary, and should go away in a few days	0=Disagree / No<br>1=Agree / Yes<br>97 = Don't know<br>99 = Refuse to answer
c) There is no need for a vaccine because COVID-19 is a conspiracy or a bioweapon	1=True / Yes<br>0=False / No<br>97 = Don't know<br>99 = Refuse to answer
d) People on the African continent are immune to COVID-19, so there is no need for a vaccine	1=True / Yes<br>0=False / No<br>97 = Don't know<br>99 = Refuse to answer
e) It is not necessary to get a COVID 19 vaccine if you follow all safety protocols	1=True / Yes<br>0=False / No<br>97 = Don't know<br>99 = Refuse to answer

f) There is not enough evidence that the COVID-19 vaccine prevents the occurrence and spread of COVID-19	1=True / Yes<br>0=False / No<br>97 = Don't know<br>99 = Refuse to answer
g) The COVID-19 vaccine was developed too fast.	1=True / Yes<br>0=False / No<br>97 = Don't know<br>99 = Refuse to answer
5.3.2. There a few different COVID-19 vaccines which have been developed in multiple countries. Does the COVID-19 vaccine's country of origin affect your willingness to take the vaccine?<br><br>*If no, skip to 5.3.4.*	0 = No<br>1 = Yes<br>97 = Don't know<br>99 = Refuse to answer
5.3.3. As of now we know that vaccines have been developed by several countries. Which countries or regions would you be willing to take a COVID-19 vaccine developed from or recommend others take?<br><br>*Read all options aloud and select all that the participant lists.*	1 = US<br>2 = China<br>3 = Russia<br>4 = India<br>5 = Europe<br>97 = Don't know<br>99 = Refuse to answer
5.3.4. If a COVID-19 vaccine had been developed or tested in Africa would that affect your willingness to take it or recommend others take it?	0=No, will not change my mind<br>1=Yes, will decrease my chances of taking it<br>1=Yes, will increase my chances of taking it<br>97 = Don't know<br>99 = Refuse to answer
5.3.5. Do any of the following groups or individuals influence your opinion about whether or not you would take the COVID-19 vaccine?	
a) Family or loved ones	0=No<br>1=Yes<br>97 = Don't know<br>99 = Refuse to answer
b) Religious leaders (imams, pastors, priests)	0=No<br>1=Yes<br>97 = Don't know<br>99 = Refuse to answer
c) Community/tribal leaders	0=No<br>1=Yes<br>97 = Don't know<br>99 = Refuse to answer
d) Political leaders	0=No<br>1=Yes<br>97 = Don't know<br>99 = Refuse to answer
e) Celebrities/social media influencers	0=No<br>1=Yes<br>97 = Don't know<br>99 = Refuse to answer
f) My colleagues / other healthcare workers	0=No<br>1=Yes<br>97 = Don't know<br>99 99 = Refuse to answer
5.3.6. Would an endorsement of the COVID-19 vaccine as safe and effective from the following actors make you more or less likely to get the vaccine?	

	a) International organizations such as WHO, UNICEF, or Gavi the vaccine alliance	1=Much more likely<br>2=More likely<br>3= No difference<br>3=Less likely<br>4=Much less likely<br>97 = Don’t know<br>99 = Refuse to answer
	b) National government or state/local government	1=Much more likely<br>2=More likely<br>3= No difference<br>3=Less likely<br>4=Much less likely<br>97 = Don’t know<br>99 = Refuse to answer
	c) The Bill & Melinda Gates Foundation	1=Much more likely<br>2=More likely<br>3= No difference<br>3=Less likely<br>4=Much less likely<br>97 = Don’t know<br>99 = Refuse to answer
	d) Africa Centres for Disease Control (Africa CDC)	1=Much more likely<br>2=More likely<br>3= No difference<br>3=Less likely<br>4=Much less likely<br>97 = Don’t know<br>99 = Refuse to answer
	e) The military	1=Much more likely<br>2=More likely<br>3= No difference<br>3=Less likely<br>4=Much less likely<br>97 = Don’t know<br>99 = Refuse to answer
	f) Other (specify)	
5.3.7.	Which of the following factors would prevent you from getting the COVID-19 vaccine, if it was available in your country?	
	a) I don't want to / cannot miss work	0=Disagree / No<br>1=Agree / Yes<br>97 = Don’t know<br>99 = Refuse to answer
	b) It is inconvenient or too far for me to travel to go get the vaccine	0=Disagree / No<br>1=Agree / Yes<br>97 = Don’t know<br>99 = Refuse to answer
	c) When the vaccine is available in my country, I will not be prioritized to get it as quickly as the wealthy/elite	0=Disagree / No<br>1=Agree / Yes<br>97 = Don’t know<br>99 = Refuse to answer
	d) I need my family consent in order to get the vaccine	0=Disagree / No<br>1=Agree / Yes<br>97 = Don’t know<br>99 = Refuse to answer

e) Other (specify)	0=Disagree / No<br>1=Agree / Yes<br>97 = Don't know<br>99 = Refuse to answer
**5.4. Information sources**	
5.4.1. Which information sources do you trust to provide accurate information about the COVID-19 vaccine?	
a) Television, radio, or newspaper	1=True / Yes<br>0=False / No<br>97 = Don't know<br>99 = Refuse to answer
b) Social media (Facebook, WhatsApp, Twitter, etc.)	1=True / Yes<br>0=False / No<br>97 = Don't know<br>99 = Refuse to answer
c) Internet	1=True / Yes<br>0=False / No<br>97 = Don't know<br>99 = Refuse to answer
d) Friends/family	1=True / Yes<br>0=False / No<br>97 = Don't know<br>99 = Refuse to answer
e) Religious bodies/leaders	1=True / Yes<br>0=False / No<br>97 = Don't know<br>99 = Refuse to answer
f) Medical professionals	1=True / Yes<br>0=False / No<br>97 = Don't know<br>99 = Refuse to answer
g) Government communications/announcements e.g. Ministry of Health	1=True / Yes<br>0=False / No<br>97 = Don't know<br>99 = Refuse to answer
h) Other (specify)	1=True / Yes<br>0=False / No<br>97 = Don't know<br>99 = Refuse to answer
**5.5. Benefits and expectations of the vaccine campaign**	
5.5.1. Would you be willing to participate in a vaccine clinical trial if one were available locally?	1=True / Yes<br>0=False / No<br>97 = Don't know<br>99 = Refuse to answer
5.5.2. When do you think a COVID-19 vaccine will be made available to you?	0=Never<br>1=Already received the vaccine<br>2=Before the end of 2021<br>3=During the first six months of 2022<br>4=During the last six months of 2022<br>5=2023 or later<br>97 = Don't know<br>99 = Refuse to answer

5.5.3.	Once vaccines are available, should people continue to follow preventative guidelines such as social distancing, washing hands, using masks, etc?	0=No<br>1=Yes<br>97 = Don't know<br>99 = Refuse to answer
**5.6.**	**Role of Healthcare Providers**	
5.6.1.	How important is it for healthcare providers to get vaccinated against COVID-19?	1=Very important<br>2=Somewhat important<br>3=Not very important<br>4=Not important at all<br>97 = Don't know<br>99 = Refuse to answer
5.6.2.	Would you recommend that your friends and loved ones get the COVID-19 vaccine?	0=No<br>1=Yes<br>97 = Don't know<br>99 = Refuse to answer
5.6.3.	Are you doing any activities to boost your community's confidence in taking the vaccine?	0=None<br>1=Community outreach/education<br>96 = Other (specify)<br>97 = Don't know<br>99 = Refuse to answer

5. Follow-up

**Question**	**Responses**
5.1.3. What is the best phone number to reach you for a future call?	

This concludes our survey. Thank you for your time and attention.
