# Supplementary material for: Perceptions and predictors of COVID-19 vaccine hesitancy among healthcare providers across five countries in sub-Saharan Africa
Source: PLOS Glob Public Health. 2025 Feb 21;5(2):e0003956. doi: 10.1371/journal.pgph.0003956 (PMC11844854; doi:10.1371/journal.pgph.0003956)
Supplement: Attachment — Submitted filename: Rebuttal letter and Response to Reviewers 9_11_23.pdf [file pgph.0003956.s005.pdf]

September 10, 2023

Editor
PLOS Global Public Health

Reference: Re-submission of Revised Research Paper for publication in PLOS Global Public Health *(*PGPH-D-22-01603R2)

Dear Editor,

Thank you for your consideration of our manuscript, "Perceptions and predictors of COVID-19 vaccine hesitancy among health care providers across five countries in sub-Saharan Africa" for publication in *PLOS Global Public Health* journal. This letter is in response to your letter dated June 20th, 2023. We would like to request a reconsideration of our article for publication in your journal.

We thank the Reviewers and Editors for a useful and comprehensive review process that started when we submitted our article on 7 October 2022. The review process was informative and helped to significantly strengthen our manuscript. We have undergone three review processes, the first with 2 Reviewers and the second and third with 6 Reviewers. In the last review, only one reviewer out of six questioned our analysis approach. Based on the majority of responses, we believe there were no serious methodological flaws with our analytical approach. One issue that was raised by the Reviewer was that we should apply the Health Belief Model in our analysis. However, our analysis was driven by a different research approach and we have included this as a limitation of our study in the discussion section. Please see the manuscript attached. Finally, our research group has used a unified analysis plan for a series of papers on vaccine hesitancy. The journal has recently published another manuscript from our research group, which has used the same analysis approach as ours.

Based on these factors, we would like to kindly ask for reconsideration. We provide a point-by-point Response to reviewers below that addresses points raised in the decision letter. We attach an updated and revised manuscript. We have also attached an article with tracked changes indicating changes made to the manuscript.

We are happy to consider any additional input that the journal may have for us. We believe that our manuscript addresses a critical issue that is under-researched in the African context. We also believe that this work contributes to the focus of the PLOS Global Public Health journal to address entrenched global inequities in public health and to highlight the work of underrepresented researchers from the African continent. We thank the Editors and the Reviewers for considering our article for publication.

Best regards,
Isabel

Isabel Madzorera, ScD
Assistant Professor, Public Health Nutrition
University of California, Berkeley | School of Public Health
2121 Berkeley Way West
Berkeley, CA 94704
imadzorera@berkeley.edu

Response to Reviewers

We thank the Editor and Reviewers for their feedback received in the decision letter. We have reviewed the comments and updated our manuscript to include the points raised. We provide a point-by-point response to the comments below.

PGPH-D-22-01603R2
Perceptions and predictors of COVID-19 vaccine hesitancy among healthcare providers across five countries in sub-Saharan Africa
PLOS Global Public Health

Dear Dr. Madzorera,

Thank you for submitting your manuscript to PLOS Global Public Health. After careful consideration, we have decided that your manuscript does not meet our criteria for publication and must therefore be rejected.
We are sorry that we cannot be more positive on this occasion. We very much appreciate your wish to present your work in one of PLOS's Open Access publications. Thank you for your support, and we hope that you will consider PLOS Global Public Health for other submissions in the future.

Yours sincerely,

Ismail Ayoade Odetokun, DVM, Ph.D.
Academic Editor
PLOS Global Public Health

Additional Editor Comments (if provided):

This manuscript still contains some fundamental flaws making it unpublishable in it's current form.

Response: We are grateful to receive comments from the Editor on this manuscript. Overall, this process has been helpful. We have revised the manuscript to address the comments received below. We hope this will be sufficient to meet the Editor’s requirements. We have also added additional information on the health belief model to clarify our thought process, and highlighted in our discussion that we used a different analytical approach, thus not using the health belief model may be a limitation. Overall, we have not received any feedback on our statistical analysis approach and believe that it meets the journal’s requirements.

[Note: HTML markup is below. Please do not edit.]

Reviewers' comments:

Reviewer's Responses to Questions

Comments to the Author

1. If the authors have adequately addressed your comments raised in a previous round of review and you feel that this manuscript is now acceptable for publication, you may indicate that here to bypass the "Comments to the Author" section, enter your conflict of interest statement in the "Confidential to Editor" section, and submit your "Accept" recommendation.

Reviewer #3: (No Response)

Reviewer #6: All comments have been addressed

---

2. Does this manuscript meet PLOS Global Public Health's publication criteria? Is the manuscript technically sound, and do the data support the conclusions? The manuscript must describe methodologically and ethically rigorous research with conclusions that are appropriately drawn based on the data presented.

Reviewer #3: Partly

Reviewer #6: Yes

Response: We are grateful for the reviewer's comments. While we are unclear on what the Reviewer's particular concerns are, we have taken the opportunity to address an issue that was raised by a previous Reviewer. The issue raised was that we should apply the Health Belief Model in our analysis. However, our analysis was driven by a different research approach and to address this we have included some literature on the health belief model in our introduction (1) and methods sections. Since we did not fully apply it in this analysis we indicate this as a limitation in our discussion section.

"One meta-analysis of studies that used the health belief model to guide the understanding of determinants of COVID-19 vaccine hesitancy found that perceived barriers and perceived benefits were most consistently associated with vaccination hesitancy (1). Other factors such as perceived susceptibility or severity, self-efficacy, and cues to action among others were informative to a lesser extent (1). However, the review did not include studies from Africa and factors influencing hesitancy may be different in the region." (Lines 139-144, page 7)

"We hypothesized that in our study context vaccine hesitancy among HCP would be informed by their perceived susceptibility, perceptions of severity of COVID-19, perceived barriers and perceived benefits, self-efficacy, cues to action and their personal characteristics. We therefore considered, COVID-19 service availability and HCP's exposure to or burden of COVID-19 patients, their knowledge levels about COVID-19 vaccines, their

perceptions of COVID-19 risk, safety, effectiveness, COVID-19 workplace practices, HCP's willingness to take COVID-19 vaccines and potential influencing factors (age, occupation, the type of health facility they work in, and their religion)." (Lines 218-223, page 11)

"Finally, our study was exploratory and descriptive. Future studies in the region should fully explore health behavior models that may explain behaviors around the uptake of COVID-19 vaccines by HCP. This will provide sufficient information to help inform policies and interventions to address this issue." (lines 550-553, page 28)

---

3. Has the statistical analysis been performed appropriately and rigorously?

Reviewer #3: No

Reviewer #6: Yes

Response: We note the Reviewer's comments. We used Poisson regression to estimate the relative risk of vaccine hesitancy. We selected Poisson over binomial regression for binary outcomes, which often suffers the problem of non-convergence. This is an accepted approach in the literature (2). In the last review, only one reviewer out of six questioned our analysis approach. Based on the majority of responses, we believe there were no serious methodological flaws with our analytical approach. Further, our research group has used a unified analysis plan for a series of papers on vaccine hesitancy. The PLOS Global Public Health journal has recently published another manuscript from our research group, which has used the same analysis approach as ours.

---

4. Have the authors made all data underlying the findings in their manuscript fully available (please refer to the Data Availability Statement at the start of the manuscript PDF file)?

The PLOS Data policy requires authors to make all data underlying the findings described in their manuscript fully available without restriction, with rare exception. The data should be provided as part of the manuscript or its supporting information, or deposited to a public repository. For example, in addition to summary statistics, the data points behind means, medians and variance measures should be available. If there are restrictions on publicly sharing data—e.g. participant privacy or use of data from a third party—those must be specified.

Reviewer #3: Yes

Reviewer #6: Yes

5. Is the manuscript presented in an intelligible fashion and written in standard English?

PLOS Global Public Health does not copyedit accepted manuscripts, so the language in submitted articles must be clear, correct, and unambiguous. Any typographical or grammatical errors should be corrected at revision, so please note any specific errors here.

Reviewer #3: No

Reviewer #6: Yes

Response: We have further revised the manuscript for flow and readability. We believe that we have adequately addressed this comment.

---

6. Review Comments to the Author

Please use the space provided to explain your answers to the questions above. You may also include additional comments for the author, including concerns about dual publication, research ethics, or publication ethics. (Please upload your review as an attachment if it exceeds 20,000 characters)

Reviewer #3: (No Response)

Reviewer #6: good work
You may do a follow up study later and see the change

Response: We think the Reviewer raises a good point. We will consider it, funding permitting.

---

7. PLOS authors have the option to publish the peer review history of their article (what does this mean?). If published, this will include your full peer review and any attached files.

Do you want your identity to be public for this peer review? If you choose "no", your identity will remain anonymous but your review may still be made public.

For information about this choice, including consent withdrawal, please see our Privacy Policy.

Reviewer #3: No

Reviewer #6: No

[NOTE: If reviewer comments were submitted as an attachment file, they will be attached to this email and accessible via the submission site. Please log into your account, locate the manuscript record, and check for the action link "View Attachments". If this link does not appear, there are no attachment files.]

In compliance with data protection regulations, you may request that we remove your personal registration details at any time. (Remove my information/details). Please contact the publication office if you have any questions.

References

1. Limbu YB, Gautam RK, Pham L. The Health Belief Model Applied to COVID-19 Vaccine Hesitancy: A Systematic Review. Vaccines (Basel). 2022;10(6).
2. Zou G. A Modified Poisson Regression Approach to Prospective Studies with Binary Data. Am J Epidemiol. 2004;159(7):702-6.
