# Supplementary material for: Perceptions and predictors of COVID-19 vaccine hesitancy among healthcare providers across five countries in sub-Saharan Africa
Source: PLOS Glob Public Health. 2025 Feb 21;5(2):e0003956. doi: 10.1371/journal.pgph.0003956 (PMC11844854; doi:10.1371/journal.pgph.0003956)
Supplement: Attachment — Submitted filename: Response to reviewers 3_28 (final).docx [file pgph.0003956.s006.docx]

**Responses to Reviewers**

5**. Review Comments to the Author**

Comment: Please use the space provided to explain your answers to the questions above. You may also include additional comments for the author, including concerns about dual publication, research ethics, or publication ethics. (Please upload your review as an attachment if it exceeds 20,000 characters)

**Response: We thank the reviewers for their suggestions and input on this manuscript. Your feedback and input have greatly improved this manuscript. We look forward to finalizing this work.**

**Reviewer #1**: Thank you for the opportunity to review this interesting and well-written paper. The paper describes a cross-sectional survey of healthcare providers in Burkina Faso, Tanzania, Ethiopia, Ghana, and Nigeria to assess their perceptions and predictors of their COVID-19 vaccination uptake behaviors and intentions.

The study introduction is framed well within the relevant existing literature of COVID-19 vaccine perceptions and uptake across countries in Africa. My overall view is that the authors have conducted an interesting study that addresses an important global health issue.

It is my opinion that this study offers important insights on in a geographical area that has been comparatively overlooked in the literature. The manuscript is well written and the proposed hypotheses and analyses are logical and compelling. I have only a few comments and suggestions for the authors.

Abstract
**Comment**:- The fourth sentence (beginning line 87), should contain information on the time period (year/months) that the survey was conducted.

**Response:** **We thank the Reviewer for this comment. We have edited the sentence to include additional information.**

**“We evaluated COVID-19 vaccine hesitancy among 1,499 HCP enrolled in a cross-sectional study conducted as a telephone survey in Burkina Faso, Ethiopia, Nigeria, Tanzania and Ghana** **between July to December of 2021.” (Lines 86-88, page 5)**

Introduction
**Comment** - Please update the case numbers since March 2022.

**Response: We have updated the papers as follows:**

**“As of March 25, 2024, there were at least 775 million cases of COVID-19 worldwide, and over 7.0 million associated deaths (1). On the African** **continent, close to 9.6 million cases and 175,503 deaths had been recorded, accounting for 2.5% of all global COVID-19 related mortality in the same period (1).” ( Lines 111-114, page 6)**

Methods
**Comment** - I am not familiar with the role of pharmacists in sub-Saharan African countries, but it is surprising that they do not have any role in delivering COVID-19 medical related services (i.e., vaccinations, testing) – could the authors provide some reference or supporting material for this statement? (line 171)

**Response: The Reviewer raises a critical point. We selected the health workers for the survey in all countries based on information received from local ARISE partners and national health associations. These informants provided information on cadres of health workers involved in providing COVID-19 medical-related services in their respective survey sites. We have added information on the selection of healthcare providers in the manuscript.**

**“****HCP were recruited through lists provided by professional associations and health facilities (2)” (Line 176, page 9)**

**Comment** - “…to meet sample requirements” (line 185). Was a sample size calculation performed? Either way, this should be discussed.

**The sample size was selected based on available resources. We had added information to clarify this.**

**“Due to resource and time constraints, the study was limited in the ability to select a larger sample.” ( Lines 201-202, page 10)**

**Comment** - “...presented in other manuscripts” (line 197). Please provide references in text.

**Response: We have added additional citations as requested by the Reviewer.**

**“The purpose of the Round 2 survey was to assess the impacts of prolonged exposure to the COVID-19 pandemic on nutrition, health, food security, and other factors among adults and adolescents in the selected countries and the findings are presented in other manuscripts (19, 22-26).” ( Lines 213-216, Page 10)**

**Comment** - I know the authors provide references to two papers (19,20) which contain further details on the ARISE network, but it would be valuable to have a brief overview of the number of survey rounds and when they occurred, this could even be in a timeline as a figure to save adding words. Or state explicitly if there are no rounds other than 1 and 2.

**Response: We thank the Reviewer for this question. There are no other survey rounds. We have clarified this in the statement below.**

**“ There were two survey rounds in the study.** **In this analysis, we used data from the second-round survey.” (Lines 210-211, Page 10)**

**Comment** - Could the authors provide a direct link to the survey questions. I followed the link on lines 166-167 but could not access them from the document.

**Response: We thank the reviewer for this suggestion. We have included the survey questionnaire under supplemental materials.**

**Comment** - The authors classified hesitancy as unvaccinated respondents who said they would definitely not, would maybe, were undecided, and were unsure. While the latter three responses indeed correspond to hesitancy, the first response seemingly better represents outright vaccine refusal (Bedford et al., 2018). Did the authors explores whether their findings differed when separating out these two groups? In the discussion the authors mention they grouped together for “ease of data analysis” (line 495), which is not particularly compelling given the potential insights that may come from exploring these two groups separately.

**Response: As a sensitivity analysis we reclassified hesitancy excluding vaccine refusal cases. When we do that the prevalence of hesitancy is 11% vs. 19.7% when refusal cases are included. When we conducted analysis to evaluate factors associated with hesitancy we found that only country, vaccine effectiveness, having numerous reasons for not vaccinating and WHO/UNICEF influence were significant predictors. The age of HCP and occupation were not significantly associated with hesitancy. We have including information from this sensitivity analysis in the manuscript.**

“**As a sensitivity analysis we reclassified hesitancy excluding vaccine refusal cases. When we conducted analysis to evaluate factors associated with hesitancy we found that only country, vaccine effectiveness, having numerous reasons for not vaccinating and WHO/UNICEF influence were significant predictors.” (Lines 481-484, page 21).**

Discussion

**Comment** - The first line of the discussion should include the time period (year/months) of the survey.

**Response: We thank the reviewer for this comment. We have added this additional information.**

**“We assessed factors influencing COVID-19 vaccine hesitancy among HCP in Burkina Faso, Ethiopia, Ghana, Nigeria and Tanzania between July to December of 2021” ( Lines 502-503, Page 24)**

**Comment** - While the authors reflect on the factors associated with vaccine hesitancy (e.g., lines 424-425) and the patterns of findings across countries, the discussion section would benefit greatly from discussion of existing efforts to address these barriers to vaccine uptake (e.g., mandates, messaging strategies, incentives). If there are no studies specifically from the relevant geographic regions the authors should highlight work done in other countries to address similar barriers to vaccine uptake and reflect on the need for their replication in this context.

**Response: We thank the reviewer for this insightful comment. We have addressed this in the Discussion section.**

**“ Given the implications for health outcomes, addressing COVID-19 hesitancy is of critical importance. Current efforts on the African continent to address this barrier to vaccine uptake could be effective if they utilize a multi-pronged approach, that includes community awareness campaigns, extensive stakeholder engagement, various communication approaches and updating government health policies and guidelines (35). A study in South Africa found that financial incentives such as cash vouchers can increase vaccination rates (36). Other strategies shown to be effective include mass vaccination campaigns in Zambia and Niger (37). Additionally, approaches such as integrating COVID-19 vaccination into routine immunization programs and primary health services could be effective (37).” (Lines 600-607, pages 27-28)**

**Reviewer #2:** The aim of this study was to describe the attitudes and behaviours of healthcare providers in 5 countries (Ethiopia, Nigeria, Burkina Faso, Ghana and Tanzania) and the factors associated with COVID-19 vaccine hesitancy. Using a cross-sectional study, the authors present a picture of the 2nd half of 2021 in sub-Saharan Africa. Many descriptive data are presented by the authors.

Abstract
**Comment:** Lines 98-99: this sentence is repetitive of the one in lines 92-93. It can be removed.
“We found higher vaccine hesitancy among HCP in Burkina Faso and Tanzania.”’

**Response: We thank the Reviewer for noting this. We have deleted the sentence on vaccine hesitancy.**

Statistical analysis
**Comment:** Line 212: It would be good to provide a table or document with all the variables, both potential confounder questions and the various questions on health behaviours or attitudes.

**Response: We have included the survey questionnaire under supplemental materials as described earlier.**

**Comment:** The choice of analysis to predict the behaviour of HCPs is not entirely clear (rational).

**Response: We note the Reviewer’s comments. We used log binomial models with Poisson distribution to estimate the relative risk of vaccine hesitancy. We selected Poisson distribution over binomial distribution for binary outcomes, as the latter often suffers the problem of non-convergence. This is an accepted approach(2). We have revised the text to address any potential confusion for the readers.**

**“We used log binomial models with Poisson distribution to evaluate associations between sociodemographic and other characteristics with vaccine hesitancy among HCPs in Round 2 of the study (3, 4).” ( Lines 237 -240, Pages 11-12)**

Results
**Comment**: There are several sections of Results that could be reduced in length. Repeating values in tables is not helpful for readers.

**Response: We thank the Reviewers for this comment. We have revised the Results section as suggested.**

Table 2

**Comment** Lines 303-305: What the n-value and % represent isn't clear. Are they the proportion of participants who answered "Yes"? It's not entirely clear what is presented in the tables in general concerning the questions on "Knowledge about COVID-19 vaccines" and "COVID-19 Prevention practices".

**Response: The reported n (percent) represent the number and proportion of HCP reporting a specified response. We have added information to clarify this in Table 2.**

Table 3
**Comment** Lines 358-360: Could "Vaccine hesitancy" be replaced by "vaccine hesitant" since we're talking about HCPs?

**Response: We have revised the term to vaccine hesitant in Table 3.**

Table 4
**Comment:** Lines 394-396: It is important to list the variables of the adjusted model under the table. “All variables included in the final model are shown in the adjusted model.”

**Response: We thank the Reviewer for this. We have included the following as a footnote to Table 4.**

**“ Variables included in the final model include country, age, respondent sex, occupation (doctor, nurse, other), health facility (government facility, private hospital, health outpost or other), religion (Catholic, none, Muslim, orthodox Christian, Protestant or other), perceived risk of COVID-19 exposure (no risk, low risk, very high risk, high risk), perceived effectiveness of the COVID-19 vaccine (not effective at all, not very effective, somewhat effective, very effective), perceived safety of COVID-19 vaccines (yes/no), COVID-19 vaccine is bioweapon (Yes/No), COVID 19 control practices (score of prevention measures being implemented in workplace (wearing masks, using personal protective equipment (PPE), hand washing with water and soap, social distancing, sanitizers or hand washing station in health facility, cleaning and decontamination or disinfection of public areas, checking temperature), yes/no), number of reasons for not vaccinating, WHO/UNICEF endorsement of COVID-19 vaccine as safe and effective affect vaccination (much more likely, more likely, no difference, less likely and much less likely).”**

Discussion
There are several questions the authors could address in the discussion:

**Comment** - What are the health policies of different countries regarding vaccination (and other vaccines)?

**Response: In the countries under review COVID-19 vaccination was promoted, with the exception of Tanzania where COVID-19 responses lagged.**

**Comment:** - What information HCPs had received during that period?

**Response: In all study countries, HCP on average had received encouragement to receive COVID-19 vaccinations and this is through COVID-19 workplace policies. While the study did not ask HCP what information they had received on COVID-19 there is evidence from the study they had access to information. For example, at least 77.6% of HCP reported workplace policies for COVID-19 and up to 84% reported that they had been prioritized as HCP to receive the vaccine. On average HCP were more likely to have access to COVID-19 vaccines as compared to the general population and vaccination rates for this group were higher than the general population . However, the exception was Tanzania, where implementation of COVID-19 policies and vaccines was delayed. However, we don’t believe that lack of information would have been a major deterrent in getting vaccination for HCP.**

**Comment**: Lines 493-494: The authors need to differentiate between vaccine hesitance and refusal (as present in the MacDonald 2015 article - Figure 1). Your choice of data to define hesitancy is problematic since you also include people who refuse, i.e. who are not hesitant. This should be reviewed.
“We defined COVID-19 hesitancy by classifying responses as, definitely not getting the vaccine, maybe, unsure, or undecided on whether to get the COVID-19 vaccine were it available.”

**Response: The Reviewer highlights a critical issue that we faced regarding the definition of vaccine hesitancy and refusal. While some authors have separated these two groups (5), others have defined vaccine hesitancy as reluctance, delay in acceptance or refusal of vaccination (6). We and others in our research group adopted the latter definition. This is also the definition used by the World Health Organization (7). There is also evidence in the literature of the use of the latter definition in the context of COVID-19 vaccines (8, 9).**

**To address issue raised by the Reviewer, we conducted a secondary analysis and added the following text to the manuscript.**

“**As a sensitivity analysis we reclassified hesitancy excluding vaccine refusal cases. When we conducted analysis to evaluate factors associated with hesitancy we found that only country, vaccine effectiveness, having numerous reasons for not vaccinating and WHO/UNICEF influence were significant predictors.” (Lines 481-484, page 21).**

**References**

1.	WHO. COVID-19 situation reports. <https://www.who.int/emergencies/diseases/novel-coronavirus-2019/situation-reports>. 2022.

2.	Assefa N, Soura A, Hemler EC, Korte ML, Wang D, Abdullahi YY, et al. COVID-19 Knowledge, Perception, Preventive Measures, Stigma, and Mental Health Among Healthcare Workers in Three Sub-Saharan African Countries: A Phone Survey. The American journal of tropical medicine and hygiene. 2021;105(2):342-50.

3.	Chen W, Qian L, Shi J, Franklin M. Comparing performance between log-binomial and robust Poisson regression models for estimating risk ratios under model misspecification. BMC Medical Research Methodology. 2018;18(1):63.

4.	Zou G. A Modified Poisson Regression Approach to Prospective Studies with Binary Data. Am J Epidemiol. 2004;159(7):702-6.

5.	Raut A, Samad A, Verma J, Kshirsagar P. Acceptance, hesitancy and refusal towards COVID-19 vaccination. (2452-0918 (Print)).

6.	MacDonald NE. Vaccine hesitancy: Definition, scope and determinants. Vaccine. 2015;33(34):4161-4.

7.	McIntosh EDG, Janda J, Ehrich JHH, Pettoello-Mantovani M, Somekh E. Vaccine Hesitancy and Refusal. The Journal of pediatrics. 2016;175:248-9.e1.

8.	Larson HJ. Defining and measuring vaccine hesitancy. Nature Human Behaviour. 2022;6(12):1609-10.

9.	Pires CA-O. Global Predictors of COVID-19 Vaccine Hesitancy: A Systematic Review. LID - 10.3390/vaccines10081349 [doi] LID - 1349. (2076-393X (Print)).
