# Supplementary material for: Perceptions and predictors of COVID-19 vaccine hesitancy among healthcare providers across five countries in sub-Saharan Africa
Source: PLOS Glob Public Health. 2025 Feb 21;5(2):e0003956. doi: 10.1371/journal.pgph.0003956 (PMC11844854; doi:10.1371/journal.pgph.0003956)
Supplement: Attachment — Submitted filename: Response to reviewers 9_23.docx [file pgph.0003956.s007.docx]

**Response to Reviewers**

**We thank the Reviewers for the feedback and guidance on the manuscript. Their comments are highly relevant and help improve our manuscript.**

**Reviewer #3: Introduction**

I appreciate the opportunity to review such a relevant manuscript, considering the challenge of collecting data on vaccine hesitancy and its implications. The research describes the perceptions and predictors of healthcare professionals in five sub-Saharan African countries in order to analyze and discuss the factors associated with vaccine hesitancy. The authors address important questions and have shown a willingness to analyze the implications and particularities of each country.

**Comment:** Line 123: In this part, the term "herd immunity" is used; I suggest replacing it with the term "community immunity". There is a global trend to avoid using terms that refer to animals, thus preventing their pejorative use in other contexts and popularizing the term community immunity.

**Response: We have revised the text to community immunity as advised (Line 123, page 6)**

**Comment:** Line 128-129: The phrase "[...] the management AND control of COVID-19 AND have a high risk of getting infected." Please replace the first "and" with a comma ",".

**Response: We have revised the text as follows,**

**“COVID-19 vaccine hesitancy among HCP could be a serious threat to efforts to combat the pandemic in Africa as they play an essential role in the management and control of COVID-19; and, have a high risk of getting infected” (Lines 126-129, pages 6-7)**

**Material and Methods**

**Comment:** Lines 188-196: As I read through, I had a question, particularly at this point: was there a criterion for selecting participants for the second round? Were the participants in the second round those who indicated vaccine hesitancy? I reviewed the methods section, and this doubt remained. Please point me to the exact description within the manuscript.

**Response: We only collected data on vaccine hesitancy in the second round of the COVID surveys. At the time of our study’s first-round surveys, there were no COVID-19 vaccines available globally. The issue of vaccine hesitancy only became relevant after the development of vaccines and their subsequent availability in the study countries. We have included the following text to clarify this.**

**“The baseline survey collected data from health professionals but did not include questions on vaccine hesitancy as COVID-19 vaccines were not available in Africa at the time of the study.” (Lines 164-166, page 9)**

**Results**

I really appreciated the tables; they were well-organized and easy to understand.

**Comment:** Table 2: I suggest splitting the table into two parts. Separate the "Frequency of availability of COVID-19 related services" from the "Knowledge about the vaccine among professionals". This would facilitate visualization and referencing of the tables throughout the results description.

**Response: As advised we have split Table 2 into two and created a new table for Knowledge about the vaccine among Health Care Providers (Table 3), page 17.**

**Comment:** Page 20: It is indicating "Figure 3: Frequent reasons (%) for not getting COVID-19 vaccination in 5 countries in sub-Saharan Africa," but Figure 2 shows the same title. Please correct the numbering.

**Response: We thank the reviewer for noticing this typo. We changed the ordering for figures previously. There is no Figure 3. We have updated the manuscript.**

**Discussion**

Some questions were answered in the discussion reading, and I appreciated how the authors addressed these findings, such as vaccinated healthcare professionals may have hesitated at some point and maintained their belief regarding COVID-19 vaccination and checking if there is any mandatory vaccination of this class.

**Comment:** I consider it pertinent that you can address more incisively the fact that nurses are among the most hesitant class along with what already exists in the literature, as nurses are on the front line and we can say that during the COVID-19 pandemic they had contact with infected people for some period at any level of health care.

**Response: The Reviewer makes an important point. We have addressed this directly in our manuscript.**

**“In this study, we found that nurses are among the most hesitant class. This is concerning given that nurses are on the front line and during the COVID-19 pandemic they had extensive contact with infected people receiving care at all levels of health systems. Vaccine hesitancy and refusal among nurses have been noted across multiple contexts ^1^. Some studies have reported that factors contributing to vaccine hesitancy among nurses include religious reasons, concerns about vaccine effectiveness, potential side effects of COVID-19 vaccines, and ethical concerns, and nurses with inaccurate knowledge about COVID-19 vaccines were likely to be hesitant, along with younger nurses ^2,3^. Our findings and those of others suggest that understanding the causes of vaccine hesitancy within this group and addressing them will be critical in future pandemics.” (Lines 541-549, pages 25-26)**

**Comment:** It was extremely important to address the study limitations, as this demonstrates that the researchers involved are attentive to data quality. I hope the study can generate a suitable impact, especially within a profession that holds authority in the healthcare field and is capable of educating health users.

**Response: We thank the reviewer for this comment. We have addressed the comments raised in the Discussion section.**

**Reviewer #4:** The paper provides important insights on the perception of HCP in SSA regarding COVID-19 immunizers. The findings are highly important for the working field. However, a few points still need to be addressed in the following section:

**Methodology:**

**Comment:** (Lines 153 and 173) The methodology section could be better structured to enhance the reader's comprehension. The subsection "study design" is presented in line 153 and repeated in line 173.

**Response: We thank the Reviewer for noting this error. We have deleted the second heading for “study design”. We have also edited the Methods section to address the reviewer’s concerns and improve the flow of the paper.**

**Comment:** (Line 159) Consider changing "study participants" to "eligible participants."

**Response: We have changed the text to “eligible participants”.**

**Comment:** (Issue with clarity of the study sampling throughout the methodology section) The authors repeatedly mention that the study was divided into two rounds. Presenting briefly the main purpose of the survey, the purpose of each round, a brief explanation of the data collected in both rounds and explaining that the paper presents data from the second round and the reasons for this at the beginning of the methodology section would make it easier to comprehend the following subsections.

**Response: We thank the Reviewer for this comment. To clarify this and avoid confusing the reader we have revised the section and added information to address the issues raised.**

**“There were two survey rounds in the study. Briefly, in the baseline survey, we collected data from HCPs in urban areas of 3 SSA countries, Burkina Faso (Ouagadougou), Ethiopia (Addis Ababa), and Nigeria (Lagos). Information on the ARISE sites in Burkina Faso, Ethiopia, and Nigeria and their characteristics are described elsewhere (19, 20). In this study, we assessed COVID-19 knowledge, perceptions, preventive measures, stigma, and mental health among HCPs. The baseline survey collected data from health professionals but did not include questions on vaccine hesitancy as COVID-19 vaccines were unavailable in Africa at the time. In the round 2 survey, data were collected from the baseline sites and additionally in rural Ghana (Kintampo) and urban Tanzania (Dar es Salaam). The purpose of the Round 2 survey was to assess the impacts of prolonged exposure to the COVID-19 pandemic on nutrition, health, food security, and other factors among adults and adolescents in the selected countries, and the findings are presented in other manuscripts (21-26). Additionally, in the Round 2 surveys, we included questions about vaccine hesitancy and were able to determine its extent and contributing factors among HCP, adults and adolescents in the selected regions of SSA. Information on the design of the Round 2 surveys is described on the Harvard University Center for African Studies website (https://africa.harvard.edu/files/african-studies/files/arise_covid_survey_round_2_methods_brief_final.pdf)..” (Lines 160-174, page 9)**

**Comment:** (Lines 171-172) The justification for the exclusion of dentists and pharmacists (and other healthcare personnel besides nurses and doctors) is still insufficient. Dentists are at high risk of acquiring COVID-19, and pharmacists are information providers for the population. The authors justify their exclusion by stating these professionals are unlikely to deliver COVID-19 medical services, but is that accurate? Clarifying that the study addressed hesitancy only in nurses and medical professionals such as physicians because those were the professionals currently available, or better clarifying the reasons for this choice, would be more suitable.

**Response: We thank the Reviewers for this comment. We do agree that two cadres, dentists and pharmacists play a critical role. However, in the African countries where we collected data, there is an acute shortage of dentists. One can go through their lifetime without ever consulting a dentist. As for pharmacists, they do provide some services but the model of health delivery is such that they are not in as close proximity to patients as doctors and nurses, and they see fewer patients. That in our view somewhat decreases their risk. This is based on reports from the local researchers and the lived experiences of the authors. We have added more information to clarify this.**

**“We excluded dentists, pharmacists, and other health providers in specialties unlikely to deliver COVID-19 related medical services in our study contexts.** **Further, doctors and nurses are available in greater numbers and have greater interface with patients in these contexts.” (Lines 188-191, page 10)**

**Comment:** (Line 173) The study sampling is quite unclear; the authors point out limitations but not how the sampling was conducted.

**Response: We thank the Reviewer for this comment. We have revised the Methods section. We hope this addresses the issues raised.**

**“****In the Round 1 study, HCP were recruited through lists provided by professional associations and health facilities in each country ^4^. Sampling frames for the study were developed using databases of HCP and their telephone numbers were provided by professional associations and health facilities in selected sites in each country. The study sites randomly selected 500 HCP to interview from the provided sampling lists in each country in Round 1 in urban areas of Ethiopia, Burkina Faso and Nigeria, with a target to recruit 300 HCP ^5^. Due to resource and time constraints, the study was limited in the ability to select a larger sample. In the new round 2 sites of Tanzania and Kintampo, HCP were recruited from medical professional associations and healthcare facilities in urban Dar es Salaam and rural Kintampo. In all sites, the inclusion criteria were HCPs currently working in a healthcare setting, inclusive of all types of health facilities where COVID-19-related services were provided. (Lines 176-188, pages 9-10)**

**Comment:** (Lines 204-207) Even though the authors point to this as a limitation later in the paper, the concept of vaccination hesitancy is an issue throughout the manuscript. The concept referenced is the vaccination scope of the WHO from 2015, which seems more related to the willingness to get vaccinated, as HCP who were vaccinated were classified as non-hesitant. However, the vaccination hesitancy scope of 2015 includes acceptance with doubts, which was not assessed in the paper. Additionally, refusal is also included in the scope and was excluded from further analysis. The new concept from 2022 ("Vaccine hesitancy” is part of the Motivation domain and defined as a motivational state of being conflicted about, or opposed to, getting vaccinated; this includes intentions and willingness") could be assessed, but classifying HCP who got the vaccine with doubts would still be an issue. Maybe instead of hesitancy, assess willingness in non-vaccinated HCP to minimize the conceptual issue, as throughout the discussion, the authors mention willingness.

**Response: We thank the Reviewers for these thoughtful comments. We agree with the suggestions made. However, at the time of the study design and implementation, we applied the WHO 2015 guidance and defined hesitancy in line with the knowledge and norms at that time and presented in other scientific work**. **We designed 3 studies in this assessment, two of which have been published using the criteria we used in this paper, including one in this same journal ^6^. We did not collect information on acceptance with doubts as outlined by the authors. We determined a priori to be consistent in the methodology and analyses conducted in all 3 papers. We are therefore limited by the data and original study design to deviate from the original research question. However, we think the Reviewer raises critical points. We acknowledge these limitations of our manuscript. If we have the opportunity to conduct similar research in the future, we will consider the recommendations made.**

**Comment:** (Collected data) As the study collected many variables, I suggest pointing out the variables collected as they are presented in the subsections of the results (for example: socio-demographic variables: follow variables presented in Table 1, and so on) to make it easier to comprehend the results section and the collected variables.

**Response: We have addressed this in the Methods section.**

**Comment:** Lastly, the results are concerning to the study field, and the study is extremely interesting and important. Just a few adjustments are still needed. I congratulate the authors on the great work!

**Response: We thank the Reviewer for this feedback and their comments. They have been informative.**

**References**

1	Khubchandani, J., Bustos, E., Chowdhury, S., Biswas, N. & Keller, T. COVID-19 Vaccine Refusal among Nurses Worldwide: Review of Trends and Predictors. *Vaccines* **10**, 230 (2022).

2	Rich-Edwards, J. W. *et al.* COVID-19 Vaccine Uptake and Factors Affecting Hesitancy Among US Nurses, March-June 2021.

3	Roberts, L. R. *et al.* Original Research: COVID-19 Vaccine Hesitancy Among Southern California Nurses. *AJN The American Journal of Nursing* **122** (2022).

4	Assefa, N. *et al.* COVID-19 Knowledge, Perception, Preventive Measures, Stigma, and Mental Health Among Healthcare Workers in Three Sub-Saharan African Countries: A Phone Survey. *The American journal of tropical medicine and hygiene* **105**, 342-350 (2021). <https://doi.org:10.4269/ajtmh.20-1621>

5	Hemler, E. C. *et al.* Design and Field Methods of the ARISE Network COVID-19 Rapid Monitoring Survey. *The American journal of tropical medicine and hygiene* **105**, 310-322 (2021). <https://doi.org:10.4269/ajtmh.20-1618>

6	Wang, D. *et al.* COVID-19 vaccine hesitancy and its determinants among sub-Saharan African adolescents. *PLOS Global Public Health* **2**, e0000611 (2022). <https://doi.org:10.1371/journal.pgph.0000611>
